# 2-Amino­cyclo­hexan-1-aminium thio­cyanate

**DOI:** 10.1107/S1600536812020879

**Published:** 2012-05-16

**Authors:** Halima F. Salem, Siti Aishah Hasbullah, Bohari M. Yamin

**Affiliations:** a School of Chemical Sciences and Food Technology, Universiti Kebangsaan Malaysia, UKM 43500 Bangi Selangor, Malaysia

## Abstract

The title compound, C_6_H_15_N_2_^+^·NCS^−^, was obtained unexpectedly from the reaction mixture of benzoyl chloride, ammonium thio­cyanate and cyclo­hexane-1,2-diamine. The cyclo­hexane ring adopts a chair conformation. In the crystal, N—H⋯S and N—H⋯N inter­actions involving the thio­cyanate anion and both the amine and the aminium N atoms link the mol­ecules, forming two-dimensional networks parallel to (001).

## Related literature

For a description of the Cambridge Structural Database, see: Allen (2002 ▶). For related thio­cyanate structures, see: Selvakumaran *et al.* (2011 ▶); Khawar Rauf *et al.* (2008 ▶). 
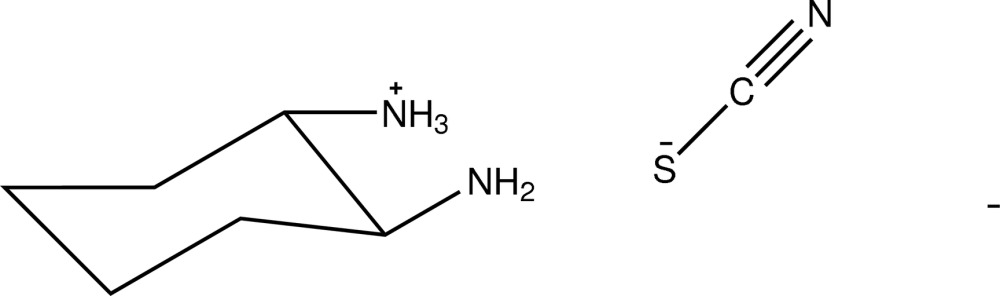


## Experimental

### 

#### Crystal data

C_6_H_15_N_2_^+^·NCS^−^
*M* *_r_* = 173.28
Orthorhombic, 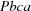

*a* = 8.590 (3) Å
*b* = 12.885 (5) Å
*c* = 17.237 (7) Å
*V* = 1907.8 (13) Å^3^
*Z* = 8
Mo *K*α radiation
μ = 0.29 mm^−1^
*T* = 298 K
0.50 × 0.50 × 0.25 mm

#### Data collection

Bruker SMART APEX CCD area-detector diffractometer
Absorption correction: multi-scan (*SADABS*; Bruker, 2000 ▶) *T*_min_ = 0.870, *T*_max_ = 0.932
10172 measured reflections
1685 independent reflections
1449 reflections with *I* > 2σ(*I*)
*R*_int_ = 0.028

#### Refinement

*R*[*F* ^2^ > 2σ(*F* ^2^)] = 0.045
*wR*(*F* ^2^) = 0.112
*S* = 1.14
1685 reflections
100 parameters
H-atom parameters constrained
Δρ_max_ = 0.25 e Å^−3^
Δρ_min_ = −0.16 e Å^−3^

### 

Data collection: *SMART* (Bruker,2000 ▶); cell refinement: *SAINT* (Bruker, 2000 ▶); data reduction: *SAINT*; program(s) used to solve structure: *SHELXTL* (Sheldrick, 2008 ▶); program(s) used to refine structure: *SHELXTL*; molecular graphics: *PLATON* (Spek, 2009 ▶); software used to prepare material for publication: *SHELXTL*, *PARST* (Nardelli, 1995 ▶) and *PLATON*.

## Supplementary Material

Crystal structure: contains datablock(s) global, I. DOI: 10.1107/S1600536812020879/lr2062sup1.cif

Structure factors: contains datablock(s) I. DOI: 10.1107/S1600536812020879/lr2062Isup2.hkl

Supplementary material file. DOI: 10.1107/S1600536812020879/lr2062Isup3.cml

Additional supplementary materials:  crystallographic information; 3D view; checkCIF report

## Figures and Tables

**Table 1:** Hydrogen-bond geometry (Å, °)

*D*—H⋯*A*	*D*—H	H⋯*A*	*D*⋯*A*	*D*—H⋯*A*
N1—H1*A*⋯S1^i^	0.87	2.53	3.3914 (19)	172
N1—H1*B*⋯N3^ii^	0.82	2.10	2.895 (3)	166
N1—H1*C*⋯N2^iii^	1.01	1.83	2.841 (2)	175
N2—H2*A*⋯N3^ii^	0.99	2.31	3.231 (3)	155
N2—H2*B*⋯S1	0.97	2.81	3.681 (2)	149

Symmetry codes: (i) 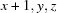; (ii) 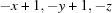; (iii) 
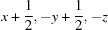
.

**Fig. 1.**
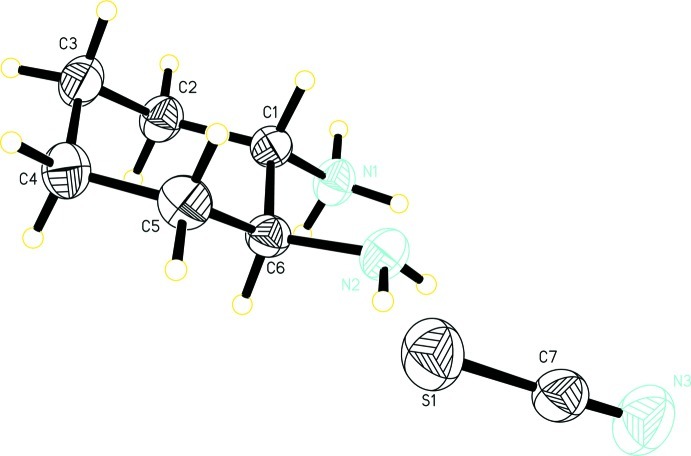
The molecular structure of (I), with displacement ellipsods drawn at the 50% probability level.

**Fig. 2.**
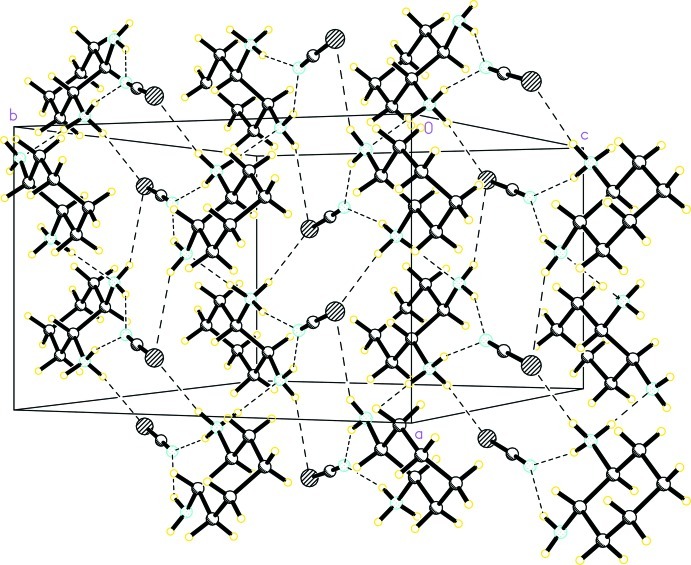
Molecular packing of (I) viewed down c axis. The dashed lines indicate intermolecular hydrogen bonds.

## supplementary crystallographic information

### Comment

The thiocyanate salts such as ammonium, potassium and sodium thiocyanate are useful reagents for organic synthesis specially for the formation of thiourea moiety. There are also some organic salts of thiocyanate such as dicyclohexylammonium thiocyanate which formed polymorph with orthorhombic (Khawar Rauf *et al.*, 2008) and monoclinic (Selvakumaran *et al.*, 2011) system respectively. Both salts were obtained rather unexpectedly from the mixture of benzoyl chloride, KSCN and dicyclohexylamine in the first and similarly, in the latter when isopthaloyl dichloride was used instead of benzoyl chloride. The title compound is analogous to the said compounds except the cation is a cyclohexane ring having a protonated and unprotonated amines at 1 and 2 positions respectively (Fig.1). The thiocyanate is linear with N3—C7—S1 bond angle of 178.22 (19)°. The cyclohexane ring adopts a chair conformation. The bond lengths and angles are in normal ranges (Allen, 2002). In the crystal structure, the molecules are linked by N1–H1A···S1, N1–H1B···N3, N1–H1C···N2 ,N2–H2A···N3 and N2–H2B···S1 intermolecular hydrogen bonds (symmetry codes as shown in Table 1) to form two-dimensional network (Fig. 2) parallel to (001).

### Experimental

All solvents and chemicals were of analytical grade and were used without purification. The mixture of benzoyl chloride (1.41 g, 0.01 mol), ammonium thiocyanate (0.76 g, 0.01 mol) and 1,2-diaminocyclohexane (1.14 g, 0.01 mol) in acetone was refluxed for 1 h. After cooling the solution was filtered and left to evaporate at room temperature. Some good crystals were obtained after 5 days of evaporation. (Yield 82%, m.p 395.9- 397.1 K). IR, NH: 3435.2, 3184.3 cm^-1^, C—N—S: 2058 cm^-1^, C—N: 1459 cm^-1^; CHNS, expt C: 48.22%, N: 24.50%, H: 8.73%, S: 17.50%), Calc C: 48.57, N: 24.20, H: 8.67, S: 18.49).

### Refinement

All H atoms attached to C atoms were fixed geometrically and treated as riding with C—H= 0.97 Å (for CH_2_) and 0.98 Å (for CH) with *U*_iso_(H)= 1.2*U*_eq_(C). The hydrogen atoms attached to nitrogen atoms were located from difference maps and refined using a riding model with *U*_iso_(H)= 1.2*U*_eq_(N).

### Crystal data

**Table:**

C_6_H_15_N_2_^+^·NCS^−^	*F*(000) = 752
*M**_r_* = 173.28	*D*_x_ = 1.207 Mg m^−^^3^
Orthorhombic, *P**b**c**a*	Mo *K*α radiation, λ = 0.71073 Å
Hall symbol: -P 2ac 2ab	Cell parameters from 2210 reflections
*a* = 8.590 (3) Å	θ = 3.0–25.0°
*b* = 12.885 (5) Å	µ = 0.29 mm^−^^1^
*c* = 17.237 (7) Å	*T* = 298 K
*V* = 1907.8 (13) Å^3^	Block, colourless
*Z* = 8	0.50 × 0.50 × 0.25 mm

### Data collection

**Table:**

Bruker SMART APEX CCD area-detector diffractometer	1685 independent reflections
Radiation source: fine-focus sealed tube	1449 reflections with *I* > 2σ(*I*)
Graphite monochromator	*R*_int_ = 0.028
Detector resolution: 83.66 pixels mm^-1^	θ_max_ = 25.0°, θ_min_ = 3.0°
ω scan	*h* = −10→10
Absorption correction: multi-scan (*SADABS*; Bruker, 2000)	*k* = −15→12
*T*_min_ = 0.870, *T*_max_ = 0.932	*l* = −20→18
10172 measured reflections	

### Refinement

**Table:**

Refinement on *F*^2^	Primary atom site location: structure-invariant direct methods
Least-squares matrix: full	Secondary atom site location: difference Fourier map
*R*[*F*^2^ > 2σ(*F*^2^)] = 0.045	Hydrogen site location: inferred from neighbouring sites
*wR*(*F*^2^) = 0.112	H-atom parameters constrained
*S* = 1.14	*w* = 1/[σ^2^(*F*_o_^2^) + (0.051*P*)^2^ + 0.6001*P*] where *P* = (*F*_o_^2^ + 2*F*_c_^2^)/3
1685 reflections	(Δ/σ)_max_ = 0.001
100 parameters	Δρ_max_ = 0.25 e Å^−^^3^
0 restraints	Δρ_min_ = −0.16 e Å^−^^3^

### Special details

**Table:**

Geometry. All esds (except the esd in the dihedral angle between two l.s. planes) are estimated using the full covariance matrix. The cell esds are taken into account individually in the estimation of esds in distances, angles and torsion angles; correlations between esds in cell parameters are only used when they are defined by crystal symmetry. An approximate (isotropic) treatment of cell esds is used for estimating esds involving l.s. planes.
Refinement. Refinement of *F*^2^ against ALL reflections. The weighted *R*-factor *wR* and goodness of fit *S* are based on *F*^2^, conventional *R*-factors *R* are based on *F*, with *F* set to zero for negative *F*^2^. The threshold expression of *F*^2^ > σ(*F*^2^) is used only for calculating *R*-factors(gt) *etc*. and is not relevant to the choice of reflections for refinement. *R*-factors based on *F*^2^ are statistically about twice as large as those based on *F*, and *R*- factors based on ALL data will be even larger.

### Fractional atomic coordinates and isotropic or equivalent isotropic displacement parameters (Å^2^)

**Table:**

	*x*	*y*	*z*	*U*_iso_*/*U*_eq_	
N1	0.87938 (18)	0.27887 (13)	0.03084 (9)	0.0437 (4)	
H1A	0.9431	0.3073	0.0637	0.052*	
H1B	0.8343	0.3242	0.0065	0.052*	
H1C	0.9375	0.2357	−0.0090	0.052*	
N2	0.55776 (18)	0.33551 (13)	0.07851 (9)	0.0436 (4)	
H2A	0.6130	0.3926	0.0516	0.052*	
H2B	0.4757	0.3638	0.1112	0.052*	
C1	0.7706 (2)	0.20856 (14)	0.07357 (10)	0.0354 (4)	
H1D	0.7077	0.1708	0.0355	0.043*	
C2	0.8674 (2)	0.13050 (15)	0.11858 (12)	0.0433 (5)	
H2C	0.9300	0.0902	0.0827	0.052*	
H2D	0.9374	0.1671	0.1532	0.052*	
C3	0.7648 (3)	0.05792 (16)	0.16548 (12)	0.0493 (5)	
H3A	0.7022	0.0162	0.1305	0.059*	
H3B	0.8297	0.0114	0.1957	0.059*	
C4	0.6591 (3)	0.11804 (17)	0.21932 (12)	0.0513 (5)	
H4A	0.7213	0.1539	0.2579	0.062*	
H4B	0.5904	0.0703	0.2462	0.062*	
C5	0.5629 (2)	0.19625 (16)	0.17426 (11)	0.0463 (5)	
H5A	0.5010	0.2366	0.2104	0.056*	
H5B	0.4919	0.1593	0.1403	0.056*	
C6	0.6618 (2)	0.26985 (14)	0.12574 (10)	0.0371 (4)	
H6A	0.7235	0.3141	0.1603	0.045*	
S1	0.15812 (8)	0.37823 (5)	0.14873 (3)	0.0610 (2)	
N3	0.2362 (3)	0.53658 (16)	0.04577 (11)	0.0678 (6)	
C7	0.2023 (2)	0.47221 (16)	0.08883 (11)	0.0443 (5)	

### Atomic displacement parameters (Å^2^)

**Table:**

	*U*^11^	*U*^22^	*U*^33^	*U*^12^	*U*^13^	*U*^23^
N1	0.0428 (9)	0.0436 (9)	0.0446 (9)	0.0029 (7)	0.0051 (7)	0.0088 (7)
N2	0.0366 (8)	0.0421 (9)	0.0520 (10)	0.0063 (7)	0.0000 (7)	0.0027 (7)
C1	0.0349 (9)	0.0353 (10)	0.0361 (9)	−0.0039 (8)	0.0002 (7)	0.0009 (8)
C2	0.0421 (11)	0.0409 (11)	0.0469 (11)	0.0055 (9)	0.0019 (9)	0.0032 (9)
C3	0.0586 (13)	0.0400 (11)	0.0493 (11)	0.0026 (10)	0.0020 (10)	0.0085 (9)
C4	0.0582 (13)	0.0522 (13)	0.0435 (11)	−0.0042 (10)	0.0079 (10)	0.0075 (9)
C5	0.0408 (10)	0.0531 (12)	0.0451 (10)	−0.0008 (9)	0.0078 (8)	−0.0006 (9)
C6	0.0366 (10)	0.0366 (10)	0.0383 (9)	−0.0002 (8)	−0.0025 (8)	−0.0029 (8)
S1	0.0708 (4)	0.0589 (4)	0.0535 (4)	−0.0088 (3)	0.0002 (3)	0.0116 (3)
N3	0.0815 (15)	0.0521 (12)	0.0697 (12)	0.0012 (11)	0.0043 (11)	0.0167 (11)
C7	0.0420 (11)	0.0442 (12)	0.0467 (11)	0.0070 (9)	−0.0023 (9)	−0.0063 (10)

### Geometric parameters (Å, º)

**Table:**

N1—C1	1.495 (2)	C3—C4	1.512 (3)
N1—H1A	0.8682	C3—H3A	0.9700
N1—H1B	0.8173	C3—H3B	0.9700
N1—H1C	1.0147	C4—C5	1.517 (3)
N2—C6	1.475 (2)	C4—H4A	0.9700
N2—H2A	0.9911	C4—H4B	0.9700
N2—H2B	0.9740	C5—C6	1.523 (3)
C1—C2	1.518 (3)	C5—H5A	0.9700
C1—C6	1.519 (2)	C5—H5B	0.9700
C1—H1D	0.9800	C6—H6A	0.9800
C2—C3	1.518 (3)	S1—C7	1.636 (2)
C2—H2C	0.9700	N3—C7	1.150 (3)
C2—H2D	0.9700		
			
C1—N1—H1A	109.2	C2—C3—H3A	109.4
C1—N1—H1B	112.9	C4—C3—H3B	109.4
H1A—N1—H1B	109.4	C2—C3—H3B	109.4
C1—N1—H1C	108.0	H3A—C3—H3B	108.0
H1A—N1—H1C	111.2	C3—C4—C5	110.68 (17)
H1B—N1—H1C	106.1	C3—C4—H4A	109.5
C6—N2—H2A	113.2	C5—C4—H4A	109.5
C6—N2—H2B	109.5	C3—C4—H4B	109.5
H2A—N2—H2B	109.9	C5—C4—H4B	109.5
N1—C1—C2	108.11 (15)	H4A—C4—H4B	108.1
N1—C1—C6	111.17 (15)	C4—C5—C6	113.01 (16)
C2—C1—C6	112.27 (15)	C4—C5—H5A	109.0
N1—C1—H1D	108.4	C6—C5—H5A	109.0
C2—C1—H1D	108.4	C4—C5—H5B	109.0
C6—C1—H1D	108.4	C6—C5—H5B	109.0
C3—C2—C1	111.24 (17)	H5A—C5—H5B	107.8
C3—C2—H2C	109.4	N2—C6—C1	110.14 (15)
C1—C2—H2C	109.4	N2—C6—C5	108.79 (15)
C3—C2—H2D	109.4	C1—C6—C5	110.16 (15)
C1—C2—H2D	109.4	N2—C6—H6A	109.2
H2C—C2—H2D	108.0	C1—C6—H6A	109.2
C4—C3—C2	111.09 (17)	C5—C6—H6A	109.2
C4—C3—H3A	109.4	N3—C7—S1	178.2 (2)
			
N1—C1—C2—C3	178.41 (15)	C2—C1—C6—N2	−173.39 (15)
C6—C1—C2—C3	55.4 (2)	N1—C1—C6—C5	−174.63 (15)
C1—C2—C3—C4	−56.1 (2)	C2—C1—C6—C5	−53.4 (2)
C2—C3—C4—C5	55.6 (2)	C4—C5—C6—N2	174.46 (16)
C3—C4—C5—C6	−55.2 (2)	C4—C5—C6—C1	53.6 (2)
N1—C1—C6—N2	65.36 (19)		

### Hydrogen-bond geometry (Å, º)

**Table:**

*D*—H···*A*	*D*—H	H···*A*	*D*···*A*	*D*—H···*A*
N1—H1*A*···S1^i^	0.87	2.53	3.3914 (19)	172
N1—H1*B*···N3^ii^	0.82	2.10	2.895 (3)	166
N1—H1*C*···N2^iii^	1.01	1.83	2.841 (2)	175
N2—H2*A*···N3^ii^	0.99	2.31	3.231 (3)	155
N2—H2*B*···S1	0.97	2.81	3.681 (2)	149

Symmetry codes: (i) *x*+1, *y*, *z*; (ii) −*x*+1, −*y*+1, −*z*; (iii) *x*+1/2, −*y*+1/2, −*z*.
